# Mechanisms of Ras Membrane Organization and Signaling: Ras Rocks Again

**DOI:** 10.3390/biom10111522

**Published:** 2020-11-06

**Authors:** Daniel Abankwa, Alemayehu A. Gorfe

**Affiliations:** 1 Cancer Cell Biology and Drug Discovery Group, Department of Life Sciences and Medicine, University of Luxembourg, Esch-sur-Alzette 4362, Luxembourg; 2 Department of Integrative Biology and Pharmacology, McGovern Medical School, University of Texas Health Science Center at Houston, 6431 Fannin St., Houston, TX 77030, USA

**Keywords:** Ras, nanocluster, Raf, dimerization, Ras orientation, membrane, HVR, structure

## Abstract

Ras is the most frequently mutated oncogene and recent drug development efforts have spurred significant new research interest. Here we review progress toward understanding how Ras functions in nanoscale, proteo-lipid signaling complexes on the plasma membrane, called nanoclusters. We discuss how G-domain reorientation is plausibly linked to Ras-nanoclustering and -dimerization. We then look at how these mechanistic features could cooperate in the engagement and activation of RAF by Ras. Moreover, we show how this structural information can be integrated with microscopy data that provide nanoscale resolution in cell biological experiments. Synthesizing the available data, we propose to distinguish between two types of Ras nanoclusters, an active, immobile RAF-dependent type and an inactive/neutral membrane anchor-dependent. We conclude that it is possible that Ras reorientation enables dynamic Ras dimerization while the whole Ras/RAF complex transits into an active state. These transient di/oligomer interfaces of Ras may be amenable to pharmacological intervention. We close by highlighting a number of open questions including whether all effectors form active nanoclusters and whether there is an isoform specific composition of Ras nanocluster.

## 1. A Brief History of Ras Nanocluster

Ras proteins are small GTPases that are critical for central cellular signaling pathways such as MAPK and PI3K/mTORC1 pathway initiation, thus driving cell proliferation, differentiation and growth. GTP-binding to Ras enables a critical conformational change primarily in its switch I and II regions [1]. This is a prerequisite for Ras to engage its downstream effectors, such as RAF, which initiates the MAPK cascade, or PI3K, which kicks-off the mTORC1 pathway [2]. Effectors possess a conserved Ras binding domain (RBD) or the structurally related Ras association (RA) domain that specifically recognize the active conformation of Ras [3].

These fundamental structural data on Ras activity were obtained by studying the soluble globular domain (G-domain) of Ras, which can be further subdivided into an N-terminal effector lobe and a C-terminal allosteric lobe [4]. By contrast, the structure of the approximately 20 residue-long C-terminal extension, called the hypervariable region (HVR), has been resolved only recently [5]. However, it is the HVR that encodes significant differences among the Ras isoforms H-Ras, N-Ras, K-Ras4A and K-Ras4B [6]. These four proteins are typically considered when talking about ‘Ras’, because of the major research focus placed on them due to their high mutation rates in cancer and developmental diseases called RASopathies [7,8]. The HVR is post-translationally lipid modified to anchor Ras to cellular membranes, and recent studies have corrected the long-held belief of the HVR as merely a hydrophobic attachment point of Ras. Membrane anchorage of Ras is necessary as Ras is otherwise unable to meet critical interaction partners for signal transmission [9]. Importantly, this domain organization is preserved amongst most of the approximately 150 human small GTPases [10], thus making Ras a heavily studied representative or ‘role model’ for other small GTPases.

As of the beginning of the millennium, the membrane raft hypothesis was intensively investigated. The hypothesis suggested nanoscale islands of specialized lipid domains that float on the highly heterogenous plasma membrane like rafts and organize signaling proteins [11]. John Hancock and colleagues provided one of the first pieces of evidence for such signaling rafts, showing that Ras proteins are non-randomly distributed in the plasma membrane, and that these so-called nanoclusters of Ras are necessary for Ras/MAPK signal output [12,13]. Based on their initial findings, they defined essential hallmarks of a Ras nanocluster [14,15]:

- Ras isoforms are laterally segregated into distinct nanoclusters
- nanoclusters have a radius of approximately 9 nm and contain up to 6–7 Ras proteins per cluster
- the average lifetimes of nanoclusters vary between 0.1 (inactive) and 1 s (active)
- approximately 40% of Ras are in immobile nanoclusters

However, many questions revolving around the actual structural changes in the clustered proteins remain unanswered.

**Figure 1:**
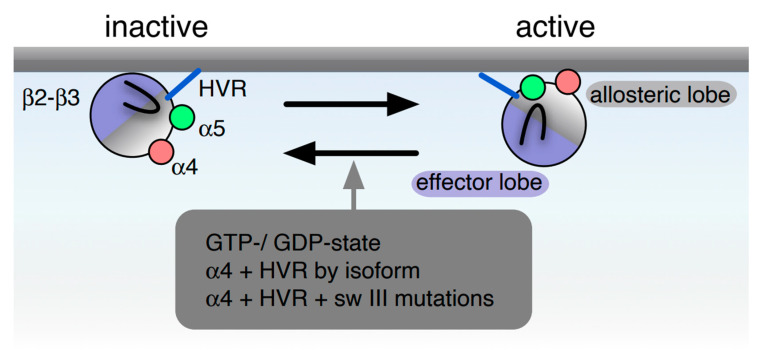
Isoform- and activation-state dependent Ras reorientation on the plasma membrane. Computational and experimental data have provided support for distinct orientation states of Ras on the membrane. Earlier work showed that in the inactive (GDP-bound) state H-Ras contacts the membrane mostly via the lipid modified HVR (blue). However, in the active (GTP-bound) state the dominant conformations have helix *α*4 (pink) contacting the membrane. Follow up work further suggested that properties of helix *α*4 and the HVR can shift the reorientation balance. As described previously, switch III (sw III) comprises the *β*2-*β*3 loop (black loop) together with helix *α*5 (green) [21].

Focusing on these issues, here we revise and extend our 2008 perspective where we integrated extant findings on nanoclustering and the then novel concept of Ras reorientation on the membrane [16] in order to derive a working model that could help to understand Ras isoform specificity [17] (Figure 1). Since then, substantial experimental proof has emerged for the existence and significance of distinct orientation states of membrane-bound Ras. Moreover, Ras nanoclustering has now come a long way from its initial definition as a cell biological observable to recent structural insights suggesting that they contain at their core multiple proteins structured around di/oligomeric units of Ras [18,19,20]. As we will discuss further below, it is plausible that one or two predominant dimer interfaces of Ras may exist that are at least transiently involved in this process. Since Ras dimerization is reviewed elsewhere in this issue, here we consider dimerization primarily in the context of its involvement in Ras reorientation on the membrane.

Existing cell biological information, in particular from single molecule tracking studies that capture Ras’s diffusional dynamics, is only incompletely integrated into the emerging structural concepts of Ras nanocluster. We have attempted to tackle these issues by also reviewing quantitative and high-resolution microscopy data gathered on intact and living cells that are at the interface of biochemical signaling studies and structural biology. Furthermore, less attention has been paid to how models that are often created for K-Ras4B (hereafter K-Ras) might apply to other Ras proteins or lipidated small GTPases more broadly. We concede that it will not be possible to reconcile all existing data. Therefore, we apply some plausibility considerations to derive a new working model of how a signaling-competent, active Ras nanocluster operates.

## 2. Zooming into a Ras Nanocluster—Insight from Electron Microscopy (EM), Single Molecule Imaging, and Förster Resonance Energy Transfer (FRET) Studies

### 2.1. Ras in the Plasma Membrane

While at steady state Ras is predominantly localized on the plasma membrane, it is constantly on the move. Its lateral mobility in the membrane (D = 1–3.5 µm^2^/s) is almost as high as that of free lipids, allowing it to diffuse across 10 µm (approximately the diameter of a small mammalian cell, and 1000-times the size of Ras) within 50 s [22,23]. Importantly, it is now well established that the steady plasma membrane localization requires directed vesicular transport of Ras to the plasma membrane [24,25]. Without this transport, even the dually palmitoylated H-Ras would undergo rapid, diffusive redistribution across all cellular membranes within seconds [26].

Visible appearance of Ras on other organelles results from regulated release or trapping of the small pool of cytoplasmic Ras. For example, a ubiquitous depalmitoylation and localized repalmitoylation on the Golgi with subsequent directional vesicular transport to the plasma membrane is necessary for the native distribution of palmitoylated Ras isoforms [26]. In the cytoplasm, Ras utilizes trafficking chaperones, such as PDE6D/PDE*δ* and calmodulin, to shield the hydrophobic farnesyl-anchor from the aqueous environment, thus allowing for more efficient diffusion of Ras [27,28,29]. Each of these trafficking chaperone systems comes with its own selectivity and release mechanism, which may further specify where Ras isoforms can be found at different states of the cell. Some evidence suggests that Ras may still be active en route to or even be activated at its intracellular locations [30,31].

However, the mechanism of Ras inactivation by the GTPase activating protein (GAP) neurofibromin (NF1) suggests that only a relatively minor proportion of Ras is active on internal membranes. NF1 is recruited to the plasma membrane via SPRED1 [32], while SPRED1 itself is probably engaged by B-RAF and predominantly delivered to K-Ras sites [33]. Hence, effector activation and Ras inactivation appear to be tightly coupled. Furthermore, given the involvement of multiple protein interactions, several context-dependent regulation mechanisms are possible during these steps [34]. It can be concluded that, at least in this particular case, the plasma membrane is the major site of Ras activity.

### 2.2. Lipid Domains and Nanocluster

The non-random organization of Ras on the plasma membrane into nanoclusters matches with what was postulated for proteins in lipid rafts. Lipid rafts were envisaged as nanoscale membrane patches in a liquid ordered (Lo) state floating inside the bulk membrane in the liquid disordered (Ld) state [11]. While in vitro data provided evidence that Ras proteins may indeed show an isoform-specific preferential partitioning to Lo or Ld membranes [35], our current view of Ras lipid engagement has evolved beyond this binary categorization [14].

The plasma membrane of mammalian/eukaryotic cells contains thousands of lipid species [11], which is already conceptually incompatible with a simple binary categorization. Early FRET data already hinted at a higher diversity of lateral organization of Ras and other GTPases [21,36]. Recent work by the Hancock lab elegantly demonstrated that K-Ras and H-Ras are surrounded by distinct phospholipid species, with K-Ras nanoclustering and membrane anchorage requiring enrichment of specific phosphatidyl-serine (PS) species [14]. Intriguingly, they also demonstrated that active H-RasG12V (but not its minimal membrane anchor, tH, a dually palmitoylated and farnesylated heptapetide representing the C-terminus of H-Ras) can negatively affect the pool of PS that is engaged by K-Ras, thus remotely downregulating K-Ras nanoclustering [37]. This intrinsic, negative feedback from active H-Ras to K-Ras may be highly significant for Ras biology, given that opposite regulation of these two Ras isoforms is also naturally implemented downstream of the mTORC1 pathway [38]. Hancock and Gorfe groups further demonstrated that the HVR of K-Ras adopts distinct conformational states that mediate specific engagements with asymmetric PS species with one saturated and one unsaturated acyl chain [39]. This is a remarkable finding, as it was believed for a long time that the polybasic HVR of K-Ras merely acts as a charge sensor [40]. Instead, an exquisite lipid sorting specificity was uncovered, which depends on the actual position of lysine residues in the HVR sequence and the nature of the prenyl anchor [39]. These results have been supported by similar data for Rac1 [41] and may therefore suggest that the high diversity of the HVR across all small GTPases in fact encodes a preferred association with certain lipid species. This observation may apply more broadly to other lipidated membrane anchoring sequences, such as those from heterotrimeric G proteins and Src kinases, underscoring the significance and scope of these findings.

### 2.3. HVR-Mediated Non-Random Distribution and Clustering

**Figure 2:**
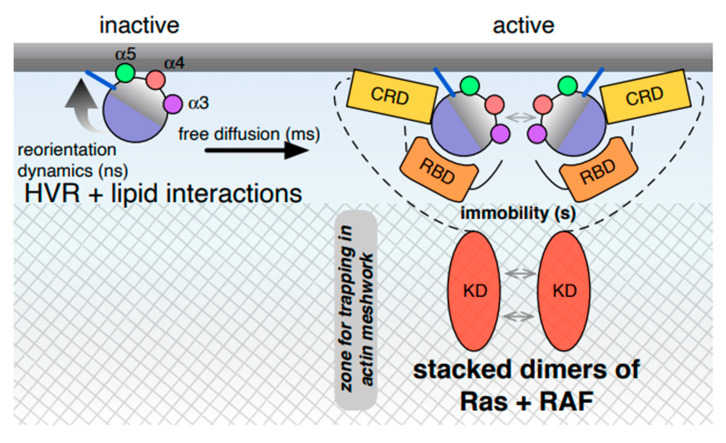
Ras dynamics on the membrane. Inactive Ras diffuses more freely than active Ras. Lateral segregation and stochastic clustering of Ras isoforms appears to be predominantly mediated by HVR interactions with membrane lipids. In living cells, diffusion can be somewhat hindered by the subcortical actin meshwork (cross hatched). Current data suggest that transient immobilization of active Ras occurs after engagement of binding partners, such as RAF effectors (domains in yellow to orange). Both increased molecular mass and enhanced trapping in the actin meshwork contribute to hindered diffusion. The minimal core of these immobile nanoclusters appears to be the dimers of Ras bound to dimers of RAF, with RAF proteins actually ‘scaffolding’ the Ras nanoclusters by enhancing trapping events. RBD—Ras binding domain, CRD—cysteine rich domain, KD—kinase domain.

The forgoing discussion suggests that specific interactions between membrane lipids and the HVR are critical for the non-random distribution of Ras on the plasma membrane. This is consistent with computational data on membrane anchors of Ras bound to relatively simple model membranes [42,43]. For example, coarse-grained molecular dynamics (MD) simulation of the H-Ras lipid anchor and its de-palmitoylated or de-farnesylated variants have shown that the nature of the lipid-modification dictates the specific lateral organization of Ras proteins, while specific lipids such as cholesterol modulate lipid domain stability and thereby nanocluster stability [42,43]. Likewise, single molecule imaging and ultrastructural data from complex cellular membranes support the non-random distribution instructed by the HVR [12,44]. However, the extent of non-randomness encoded in the HVR and how that relates to Ras di/oligomers or active nanoclusters remained unclear. Importantly, spontaneous formation of Ras dimers has been observed in cells [44], which, in light of what is explained in the next paragraph, may be referred to as HVR-induced, lipid-mediated clustering (Figure 2). When the formation of active K-Ras dimers is enforced by a chemically induced, tag-mediated dimerization system at low, near native Ras expression levels, it has a significant impact on MAPK signaling [44]. Interestingly, the non-random characteristics of K-Ras distribution in the membrane strikingly matches those of RAF-dimers with the occurrence of dimers at similar frequencies, consistent with complexes of Ras dimers paired up with RAF dimers [18,44].

However, when native (i.e., farnesylated and carboxymethylated) K-Ras was investigated by fluorescence correlation spectroscopy (FCS) and single molecule tracking on supported lipid bilayers of various lipid mixtures, no evidence for dimerization was found [22]. Yet, encounters of Ras do take place in these artificial membrane systems as it was possible to photo-crosslink H-Ras into dimers that depend on Y64 [45]. All of these membrane oligomerization data are naturally sensitive to the 2D density of the Ras construct under investigation, hence differences in the observations may partly relate to the specific experimental conditions. Yet, comparison of the cellular and in vitro data may suggest that the complexity of the cellular membrane and/or additional factors in living cells foster more stable and/or frequent dimerization that goes beyond the HVR-mediated non-random assemblies.

### 2.4. Correlation of Single Molecule Tracking Data with Ras Nanoclustering

Extensive single molecule tracking analyses by the Kusumi lab established several years ago that the actin meshwork provides for a hierarchically organized barrier (picket-fence) that restricts diffusion even of phospholipids [46,47]. Moreover, by coupling single-molecule FRET to detect Ras activation with single-molecule Ras lateral diffusion measurements in living cell membranes, the same group has established an important hallmark of Ras activation in cellular membranes [23]. Before activation, only 16% of H-Ras were immobile, while EGF stimulation increased that fraction to 55%. Very similar results were obtained by others, showing that predominantly (75%) fast diffusing H-Ras becomes more confined upon activation, with S17N and G12V mutants behaving essentially like H-Ras before and after stimulation, respectively [48]. Strikingly, the fraction of immobile Ras correlates with the fraction of nanoclustered RasG12V [15,49], suggesting that active Ras is immobilized in nanoclusters. But what is the makeup of these immobile, active nanoclusters that initiate MAPK signaling? The following two observations suggest that binding of effectors or other binding partners to Ras are intimately involved: (1) Upon recruitment to activated Ras, both the Ras binding domain of p120RasGAP and the full-length C-RAF become immobilized in a manner that is sensitive to actin-meshwork disruption [23,50]. (2) There is a striking match between the fraction of RAF molecules with a longer membrane residence time of 1.6 s (37% of RAF) and that of the aforementioned immobile/hindered Ras in the membrane [23,48], suggesting that binding of regulators or effectors to active Ras confines them in the actin-meshwork. Indeed, according to the picket-fence model, the formation of large signaling complexes would lead to the trapping of the complexes in the sub-membraneous cytoskeletal meshwork that is mostly composed of actin [47]. Therefore, the most significant change in Ras mobility occurs when molecular complexes are formed, such as by effector complex engagement, leading to prolonged transient immobilization, a feature that by correlation can be ascribed to an active nanocluster (Figure 2).

Indeed, this correlation goes beyond the similarity of the immobile and membrane-bound fractions of Ras and RAF. RAF dimer-inducing RAF inhibitors (RAFi) increase Ras nanoclustering and the fraction of immobile Ras, thus directly linking a specific structural and functional event (RAF-dimerization, which also correlates with RAF activation) with the formation of immobile, active nanoclusters [51]. Further support for the coupling of RAF dimerization and Ras nanoclustering was provided by showing that a dimeric RBD was sufficient to mimic the effect of a RAFi. Another important observation was made here, namely that RAFi-induced RAF dimerization and the ensuing increased MAPK signaling happens at the expense of PI3K signaling. Therefore, stabilization of RAF dimers on active Ras by inhibitors also blocks or diminishes access of other effectors such as PI3K [51]. The nanocluster scaffold galectin-1 (Gal1) likewise shifts H-Ras signaling to increased MAPK but decreased PI3K/Akt signaling [52,53]. Similar to RAFi, Gal1 slows down the diffusion of GTP-H-Ras in cells [49]. Together with the fact that Gal1 is a dimeric protein that directly binds the C-RAF-RBD, this observation led to a model in which Gal1 facilitates RAF dimerization to increase Ras nanoclustering [18]. Combining these observations, we propose that dimeric RAF is not only a part, but a necessary component of functionally relevant, active Ras nanoclusters that mediate MAPK signaling (Figure 2). Once RAF is bound to active Ras within nanoclusters, the 2D mobility of Ras is drastically reduced due in part to the diffusion barriers created by the sub-membraneous actin meshwork. Notice that this is different from the HVR-mediated slight mobility drop of Ras that would occur irrespective of the Ras activation state. We will return to some of these issues when discussing isoform-specific Ras membrane orientations and RAF engagement.

## 3. Activation State Dependent Orientations of Ras on the Membrane

More than a decade ago, we proposed that Ras orientation on the membrane represents another level where Ras isoform specificity and activity is defined [17]. The first evidence for different Ras orientation states on the membrane was provided by a seminal MD simulation study that suggested two distinct preferred conformational states of H-Ras, depending on whether it is GDP- or GTP-bound [16]. Two structural elements that are highly diverse amongst all Ras proteins, helix *α*4 and the HVR, stabilize the different orientations of the Ras G domain on the membrane [6,10]. Mutational analysis of these regions revealed that the resulting orientation mutants systematically modulate biological activities in a manner that is consistent with the computational predictions [16,21] (Figure 1). Importantly, the recruitment of the C-RAF-RBD varies according to the Ras orientation mutant, suggesting a direct conformational readout of the reoriented Ras by the effectors. All of these data supported a ‘balance model’ wherein the GTP-preferred orientation of Ras displayed an increased effector recruitment and MAPK output, while the opposite was true for the GDP-preferred orientation [17].

In addition, a connection between structural changes in the G-domain and the reorientation of Ras on the membrane was sought. Guided by simulations and analysis of crystal structures of Ras, specific residues in the *β*2-*β*3 loop and helix *α*5 were mutationally analyzed [21]. These two structural elements exhibit correlated motions when Ras undergoes a GTP-induced conformational change [54]. Intriguingly, the mutational analysis revealed phenotypic similarities to the orientation mutants, suggesting a coupling of reorientation with this novel ‘switch III’ region [21]. Indeed, switch III constitutes another part of the Ras structure that undergoes subtle, but significant conformational changes during Ras activation [4,54]. Conformational changes in the effector lobe of Ras, which contains switch I, II, and part of switch III (the *β*2-*β*3 loop, also known as inter-switch region), induce reorganization of the C-terminal helix *α*5 (which is part of switch III) and the adjacent HVR [4,16,54,55]. This tug on the C-terminus that originates from the nucleotide binding site is therefore likely to lead to an altered conformational balance on the membrane. Further evidence for the coupling of switch III with helix *α*4 and the HVR was provided by creating mutants where activity-increasing orientation mutations were combined with activity-decreasing switch III mutations and vice versa [56]. In support of the coupling, all of these mutants returned to baseline C-RAF-RBD recruitment activity. Intriguingly, rare cancer associated mutations in the switch III region of all Ras isoforms show increased nanoclustering, effector recruitment and MAPK-output, suggesting a biological relevance of the coupling between Ras reorientation and nanoclustering [56,57].

Following the original report on H-Ras [16], extensive MD simulations in recent years have shown that the G-domain of G12D [58], G12V [59], Q61H [60] and wild type K-Ras [61] directly interacts with membrane lipids via multiple distinct orientations. In some of these orientations, the G-domain is unable to interact with effectors because of the occlusion of the switch loops by the membrane. The simulations have also shown that the relative disposition of the HVR and the G-domain underpin membrane reorientation [58,59,60,62]. Altogether, these computational results are consistent with observations from nuclear magnetic resonance (NMR) and single frequency fluorescence anisotropy decay experiments [63,64,65]. Although orientations in which the effector binding region is solvent exposed dominate in most simulation studies, more work is needed to accurately determine the relative population of the different orientation states. This includes a state, sometimes referred to as an intermediate orientation [59,60], in which the G-domain is solvent exposed and distal from the membrane. A recent report, based primarily on neutron reflectometry (NR) analysis of K-Ras bound to a sparely tethered bilayer membrane [62], found that the membrane-distal conformation is dominant (90%). While the dominance of this conformation in cells cannot be ruled out at this point, it stands in contradiction with multiple computational and experimental studies that showed the ability of K-Ras to sample multiple distinct orientations including those that allow the G-domain to directly engage the membrane. Nonetheless, we argue below that the available data in aggregate suggest a “grab-and-swing” model that somewhat resembles the “fly-casting” mechanism of RAF recognition by K-Ras proposed by Van et al. [62].

Of note, identical mutations on helix *α*4 of H-Ras and K-Ras oppositely modulate effector recruitment [6], suggesting that the Ras reorientation balance is isoform specific and depends on the sequence and structure of helix *α*4 and the HVR (Figure 1). Some of the first independent experimental data supporting multiple distinct orientation states of Ras came from the Winter lab. They showed that, also in an in vitro system, H-Ras and K-Ras have opposite nucleotide-dependent orientations on the membrane, with GDP- and GTP-K-Ras showing more or less membrane attachment, respectively [66,67]. Indeed, analysis of additional Ras isoforms suggests a systematic variation of the membrane orientation of the G domain by the membrane association tendency of helix *α*4 as compared to that of the HVR [6,17]. Based on an elegant application of NMR to K-Ras bound on lipid nanodiscs, Mazhab-Jafari MT, et al. found that disease-causing RASopathy mutations such as K5N and D153V modulate the membrane orientation of K-Ras [65]. Combining MD and spectroscopic approaches, additional studies by the Sligar and Buck groups have established that membrane reorientation is modulated by the lipid composition of the host membrane [63,68]. Helix *α*4 and the HVR were proposed to constitute two structural elements that were used to balance the reorientation equilibrium, thus modulating the preferred orientation and probably also the ease of the reorientation [17,21]. This mechanism was proposed to be more broadly applicable to prenylated small GTPases [69]. While these data require further scrutiny, including testing for potential effects of the mutations on di/oligomer formation, they suggest a mechanism of how the naturally occurring high sequence diversity on helix *α*4 of Ras subfamily proteins and their HVR is translated into isoform-specific orientation balances on the membrane.

## 4. Why Is the Biological Activity of Different Ras Orientation Mutants Changed? Occlusion of the Effector Binding Site vs. That of the Dimer Interface

How is effector recruitment modulated by the different Ras orientations and why does it correlate with altered nanoclustering? In solution, Ras orientation mutants and switch III mutants interact identically with the C-RAF-RBD [56,57], confirming that the effector interface is not altered by the mutations. When examined in the membrane of intact cells, however, the orientation- and switch III-mutants show altered nanoclustering that correlates with C-RAF-RBD recruitment. This was demonstrated by using Gal1 to modulate nanoclustering of H-RasG12V orientation- and switch III-mutants, revealing that the membrane mobility of the more active orientation mutants was more efficiently reduced by Gal1 than that of the less active orientation mutants [57].

Another facet of the effect of Gal1 on Ras nanoclustering is that it is isoform selective [18,52]. Gal1 increases H-RasG12V and decreases K-RasG12V nanoclustering while it is neutral on N-RasG12V, with corresponding consequences for MAPK output and opposite effects on PI3K/Akt signaling [52]. How exactly does Gal1 selectively increase nanoclustering of only a specific Ras isoform if it binds to the RBD of RAF proteins? While we cannot conclusively answer this question yet, it is possible that Gal1 stabilizes certain RAF dimers that may be necessary for the nanoclustering of a given Ras isoform. This would imply that Ras isoforms and by extension Ras orientation mutants somehow select for certain RAF dimers. Alternatively, differences in dimerization profiles among Ras isoforms may result in variations in effector- and/or Gal1-recognition and thereby nanocluster stability. The latter scenario could be realized if different Ras isoforms or orientation mutants offer different interfaces for Ras dimerization.

### Ras Dimers and Their Plausibility

**Figure 3:**
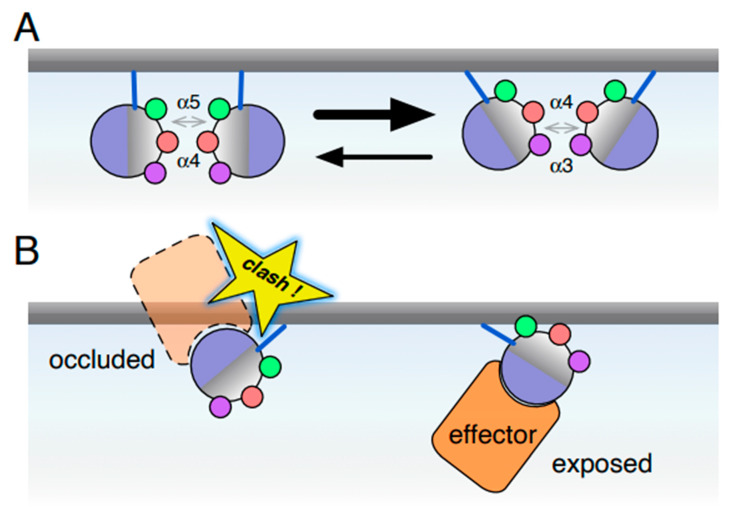
Ras dimerization and reorientation affect access to binding partners. (**A**) Several dimer interfaces for Ras have been proposed. The most prominent ones center around either helices *α*4 and *α*5 or helices *α*3 and *α*4. Dimer affinities are expected to be weak, even if Ras concentration is effectively elevated in the 2D membrane. (**B**) Reorientation of Ras on the membrane may occlude the effector lobe, thus restricting access to binding partners such as effectors. Reorientation and dimerization employ overlapping secondary structural elements, such as helix *α*4. Therefore, it can be expected that they mutually affect one another.

A number of recent reports based on in vitro and cellular data support the existence of Ras dimers. The apparent consensus is that these dimers have a very low affinity in solution (likely in the range of mM) and are held together by interfaces involving helices *α*4/*α*5 or *α*3/*α*4 plus loops connecting the helices and *β*-strands [70,71]. K-Ras dimers held together through these interfaces were predicted to be only marginally stable in solution [70,72] (Figure 3A). Given the overlap of some of the residues involved in membrane reorientation and dimerization, the dimer interfaces are unfortunately difficult to distinguish from those involved in reorientation. Nonetheless, introduction of a putative disulfide-bridge into the *α*3/*α*4 dimer interface of K-Ras was found to enhance ‘nanoclustering’ likely by increasing the number of dimers within the nanoclusters [20,70]. Additional analyses using single molecule spectroscopy in live cells and molecular modeling of K-Ras mutants predicted to stabilize or destabilize the *α*3/*α*4 interface showed that K-Ras exists as dimer, trimer, tetramer or even pentamer [20,70]. A key conclusion of this work was that the same two partially overlapping *α*4/*α*5 and *α*3/*α*4 interfaces can combine variously to form quasi-symmetric multimers whose stability is likely complemented by protein-lipid interactions involving residues at the HVR. These observations support not only the existence of weak affinity K-Ras di/oligomers in cells but also the view that stabilizing dimers increases the propensity of Ras to form nanoclusters.

Others have used a different approach to inducibly force the dimerization of GDP- and GTP-K-Ras and suggested that the resulting non-productive *α*4/*α*5 dimers explain the tumor suppressive effect of wild-type K-Ras alleles in heterozygous K-Ras mutant cancers [73]. However, in vitro NMR-based experiments suggest that *α*4/*α*5-mediated cross-dimerization of GTP- and GDP-loaded K-Ras does not occur [74]. The potential for non-functional dimers is further illustrated by the compound BI-2852, which was meant to block essentially the effector-lobe of Ras [75] but was then found to induce non-functional K-Ras dimerization [76]. It seems, therefore, worthwhile to reexamine when and where in the course of Ras activation are we dealing with a clear (druggable) dimer interface. If Ras dimer interfaces are significant for Ras activation, they should be phylogenetically conserved and the helices that are implicated in the interfaces should have co-evolved. Based on this consideration, the *α*3/*α*4 interface is somewhat more supported than the *α*4/*α*5 by results from sequence co-evolution analysis [70], which suggested that many residues on the three allosteric lobe helices and some of the effector lobe loop regions are involved in conserved interactions. On the other hand, support for the importance of the *α*4/*α*5 interface has been provided by structural and biochemical studies of a monobody, called NS1, that impairs Ras signaling by binding to the *α*4/*α*5 dimer interface and thereby presumably disrupting dimer and/or oligomer formation [77].

Further examination of the sequences of the helices involved in dimerization and/or reorientation makes it apparent that helix *α*4 is relatively similar within a clade of human Ras isoforms (H-Ras, N-Ras and K-Ras4A/B) whereas the HVR is more similar in R-Ras1, R-Ras2 and M-Ras [6]. The relative similarity of either of these regions could hint at some cross-dimerization reactivity within a clade. However, H-Ras and K-Ras, which share a nearly identical *α*4 sequence, are laterally segregated in distinct nanoclusters in order to stay functional [37]. Moreover, a chimera that had helix *α*4 of Ras replaced by the amphipathic membrane anchoring motif of the MARCKS protein behaves like orientation mutants in terms of effector recruitment [6,21]. If the Ras helix-based dimers were stable on their own, the replacement of the entire helix *α*4 with the MARCKS sequence should have seriously disrupted the dimer interface involving helix *α*4. This would suggest that functionally significant Ras dimers may not exist as a significant fraction without additional proteins that bind to them (such as RAF effectors), and the diffusion confinement imposed by the actin-based meshwork. It is possible, therefore, that dimer interfaces would transiently become relevant in complexes with effectors, as Ras fluctuates between active sub-states that may be relevant to actuate conformational changes of the effectors to fully activate them.

## 5. What Do We Know about the Activation of Raf by Ras on the Membrane?

Current evidence supports a role for RAF effectors in stabilizing Ras dimers, and thus the functionally relevant active Ras nanoclustering, with Ras reorientation possibly playing a role in selecting for specific RAF dimers. Below, we review some of the key lessons learned in the past few years regarding how RAF engages Ras, with emphasis on structural insights from a broad array of methods that have been instrumental in shaping our current understanding of Ras nanoclusters.

First, it is well accepted that RAF is in an autoinhibited state in the cytoplasm, and that it gets transiently uninhibited upon recruitment to the plasma membrane by Ras [78]. Secondly, single molecule analyses have shown that RAF proteins constantly bounce onto the membrane until they encounter an active Ras, a process likely facilitated by an HVR-mediated pre-clustering of Ras [50,79]. Thirdly, Ras binding primarily involves the RBD of Raf. However, targeting RAF to the plasma membrane by genetically fusing it to the K-Ras HVR (RAF-CAAX) is sufficient to activate RAF [79]. Because the RAF-CAAX chimera is less likely to leave the membrane as compared to its native counterpart, it can be expected that RAF-CAAX encounters active endogenous RAS with a higher probability than normal RAF. This may explain how RAF-CAAX is highly active [79]. Moreover, allosteric coupling between the N- and C-termini of RAF facilitates dimerization of the C-terminal kinase domains [80]. Thus, RBD binding and associated changes at the N-terminus facilitate kinase domain dimerization. Conversely, RAFi’s that stabilize the kinase domain dimer open up the N-terminus for increased engagement with Ras [80,81], which explains how these compounds can increase Ras nanoclustering and signal output [51].

Recent computational and biophysical experiments have provided additional details of how the RBD and the adjacent cysteine rich domain (CRD) of RAF synergize when binding to K-Ras on the membrane, whereby the CRD mediates membrane association predominantly via lipid and electrostatic interactions [82,83]. NMR experiments support such synergism, showing an increased affinity of the K-Ras/RBD-CRD complex for the membrane (K_d_ = 10 µM) as compared to that of either protein alone (K_d_ = 20–30 µM) [64]. Of note, the CRD is unable to bind K-Ras on its own. Furthermore, MD simulations suggest that the CRD reorients the RBD such that occluded orientations of Ras, which are less accessible to effectors, are less populated while the allosteric helices *α*3, *α*4 and *α*5 of Ras become available for potential dimerization [84]. Simulations of the K-Ras/B-RAF-RBD-CRD complex further revealed that the CRD influences which membrane orientation states are assumed [83]. These orientations are differently conducive to effector engagement and Ras dimerization: the exposed (effector accessible) orientation is stabilized by membrane contacts via helices *α*4/*α*5, thus making the *α*3/*α*4 interface available for dimer formation while the occluded orientations allow for both the *α*3/*α*4 and *α*4/*α*5 dimer interfaces becoming accessible to solvent. Using NMR, a third report found two distinct conformational states in the K-Ras/RBD-CRD complex [64]. The first (occluded) state is stabilized by membrane contacts from K-Ras helices *α*4 and *α*5 plus the canonical lipid binding site on the CRD (Figure 3B). Interestingly, with higher density of the complex, a second state was favored, which contacts the membrane via parts of the RBD and CRD, as well as parts of K-Ras *α*5. Notably, only the second state has interface *α*4/*α*5 available for interactions, and the E125K cancer-associated mutation in C-RAF-RBD shifts the equilibrium to this state [64].

**Figure 4:**
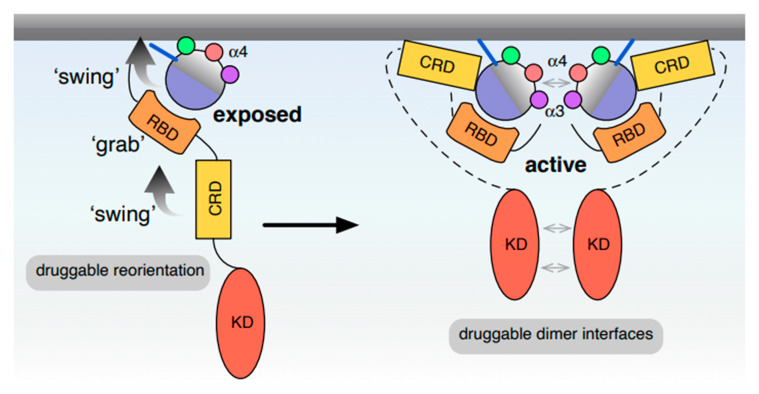
New model for Ras reorientation and RAF-dependent nanoclustering. We propose a ‘grab-and-swing’ model, where reorientation dynamics contribute to the full membrane engagement of Ras-bound binding partners such as the effector RAF (left). The rearrangement of the whole membrane bound complex is then further stabilized by at least transient dimer interfaces of Ras. Note that high-affinity, rigid Ras dimer formation could impair the activity of the whole complex, while allosteric coupling between the Ras-engaged N-terminus of RAF and its C-terminus reinforces dimerization of the kinase domain, which is required for RAF activation.

Collectively, these observations suggest that, following Ras activation and random collision of the partially accessible RAF-RBD with Ras in the membrane, the CRD engages the membrane with a lipid selectivity profile that mimics that of K-Ras [85]. Other parts of RAF may provide Ras isoform selectivity. An example would be the N-terminus of B-RAF that specifically engages the K-Ras HVR [86]. The engagement of RBD and CRD with Ras stabilizes the entire complex on the membrane and imposes local reorientation of the different protomers. We propose that conformational selection processes at this stage facilitate preferential pairing of a given Ras isoform with RAF dimers, such that certain RAF dimers are preferred over others. The activity of this pairing is determined by the preferred Ras orientation on the membrane and the ease of its conformational transitions, and by how effectively Ras engagement is allosterically transmitted from the N-terminus of RAF to its C-terminal kinase domain (Figure 4). The latter may be observed by monitoring how easily a RAF protein transits into an open state. Different Ras orientations would therefore lead to variations in the opening process or the population of the open states of RAF. While distinct binding preferences of Ras-RAF pairs were reported recently [86], the work did not explicitly take into account that RAF binds as homo- or hetero-dimer to Ras. Furthermore, KSR proteins, the RAF-like pseudokinases without an RBD, can heterodimerize with RAF proteins and modulate its activity [78].

Because Gal1 binds to the RBD of RAF and somehow recognizes the orientation of the Ras/RAF complex and affects Ras nanoclustering in an isoform specific manner [6,57], signal output may be fine-tuned by such accessory proteins. In the context of this model, Gal1 likely binds RAF that is bound to a more active, possibly more solvent exposed, orientation of Ras, enabling the open state of RAF more efficiently. Such a scenario vastly increases the complexity of MAPK signaling strength regulation and points to multiple opportunities for signal integration and modulation by other pathways. The resulting fine-tuning of MAPK signaling may not be easy to capture experimentally (e.g., by phospho-ERK Western blotting). However, given the occurrence of RASopathy mutations with subtler effects on MAPK signaling, such fine tuning is probably highly significant in physiology. Hence, phenotypic differentiation assays could be very useful to work out subtle differences in MAPK signaling [87].

A number of mutations with subtle MAPK effects have now been mechanistically linked to the above Ras-RAF activation scenario [56,64,65], highlighting that unknown conformational states are disease relevant. Consequently, interfering pharmacologically with such conformational states on the membrane may open up novel ways of modulating the Ras/MAPK activity more subtly, an intervention strategy that is needed in the case of chronic, long-term treatments such as for RASopathy patients. Proof-of-concept studies have demonstrated that it is possible to identify small molecules that can accomplish this. For instance, the small molecule Cmpd2 binds to a pocket between K-Ras and the membrane, stabilizing K-Ras in an occluded orientation [64]. Dedicated technologies such as the second harmonic generation (SHG)-based screening method are sensitive to the orientation of a protein and may therefore enable specific drug screening campaigns for modulators of the Ras/RAF conformation in the future [88] (Table 1).

## 6. Conclusions and Perspectives

Combining all of the data discussed in the previous sections, we propose that the selective engagement of a given Ras protein and an effector is determined by several factors. First, the actual surface that recognizes the effectors (via their RBD or RA domain) seems to be relatively conserved among Ras proteins within a subfamily clade [93]. Yet affinity differences between the RBD or RA and Ras can span more than three orders of magnitude [3]. This thermodynamic gate constitutes a first level of specialization for Ras-effector coupling, potentially separating distinct effectors such as RAF and PI3K. Secondly, insights from other small GTPases suggest that additional contacts of the effector outside of the switch regions are important to further define specificity [94]. Importantly, a recent crystallographic study of K-Ras in complex with the RBD-CRD domains of C-RAF found that the CRD domain directly interacts with residues in the switch III region of K-Ras [95]. Thirdly, given that the orientation changes of Ras on the membrane appear to be Ras isoform specific, we argued that it is likely that this mechanism further contributes to defining effector selectivity and engagement, possibly on the paralog level. These isoform specific orientation states are significantly modulated by residues on helix *α*4 and the HVR. Finally, the HVR sorts Ras proteins into specific lipid domains, which could impact effector selectivity considering that most effectors contain domains that interact with certain membrane lipids. All of these processes are further modulated and fine-tuned by Ras di/oligomerization, binding to dimeric effectors, lipid composition, and the actin meshwork. Considering that some of the same residues involved in membrane engagement are also involved in dimerization, we propose a “grab-and-swing” model, where reorientation dynamics contribute to the full membrane engagement of Ras-bound binding partners, such as the effector RAF (Figure 4).

Despite the substantial progress that was made in recent years, the dynamic aspects of Ras functioning, such as conformational transitions or diffusion in the membrane, are all too often neglected when conceiving molecular mechanisms because they are more difficult to acquire and interpret than structural snapshots. One of the great advancements in the last decade is the ability of computational molecular simulations to connect static structural data with dynamic processes. Furthermore, high resolution live-cell imaging techniques, such as super-resolution microscopy, Förster resonance energy transfer (FRET), single molecule tracking, and fluorescence fluctuation methods, have now become more accessible to the scientific community. These techniques, in combination with the current capabilities of computational methods, have the power to resolve molecular details at the spatiotemporal resolutions necessary to dissect Ras signaling in its full complexity.

With the help of these and other approaches, many of the outstanding questions around Ras orientation, nanoclustering and signaling may be tackled in the near future. These include: (1) Are Ras orientation states and nanoclusters the same among different cell types and throughout a cell, given that lipid content varies from cell to cell and typically also within a cell (e.g., apical vs. basolateral sides)? Differential lipid distribution should fine-tune the extent of the reorientation dynamics and nanoclustering and ensuing signal output across the cell. (2) What other functionally relevant components are there in Ras isoform-specific nanoclusters? Recent evidence suggests that prohibitin is part of a K-Ras nanocluster and can be targeted by the nanomolar inhibitor rocaglamide to potently reduce growth of KRAS mutant cancers [89]. (3) Do other effectors, such as PI3K, also stabilize active nanoclusters in a similar manner as RAF proteins? (4) Is there a relevant Ras dimer interface that can be drug-targeted? Or what are the structural changes inside a nanocluster? What kind of allosterism and cooperativity is critical? Addressing these issues will clearly advance our understanding of Ras signaling, shape our understanding of Ras associated diseases, and guide future innovative drug development efforts against the Ras signaling machinery.

## Figures and Tables

**Table 1:** Disease manifestations of critical Ras-RAF activation steps and potential inhibitors or drug targets.

Activation Step	Disease Manifestation	Example Inhibitor/Target
Ras lateral segregation of nanocluster	isoform specific Ras mutation frequency in cancer [7]	prohibitin inhibitors [89], salinomycin [90]
Ras orientation	RASopathies and cancer; mutations in Ras switch III [56,65]	Cmpd 2 [64]
Ras dimerization	unknown	NS1 monobody [77]
RBD binding	RASopathies; RBD mutations (Jindal 2015)	switch I/II pocket inhibitors (BI2852) [75,91]
RAF opening state	cancer; multiple RAF mutations	RAFi [80,81]
RAF dimerization	unknown	RAFi
RAF output activity	RASopathies and cancer; mutations all across RAF	RAFi [92]
Ras-RAF complex modulators	cancer; expression changes of modulators	galectin-1, galectin-3 [18,52]
